# The carbon footprint and energy consumption of liver transplantation

**DOI:** 10.3389/frtra.2024.1441928

**Published:** 2025-01-06

**Authors:** Paolo De Simone, Quirino Lai, Juri Ducci, Daniela Campani, Giandomenico Biancofiore

**Affiliations:** ^1^ Liver Transplant Program University of Pisa Medical School Hospital, Pisa, Italy; ^2^ Department of Surgical, Medical, Biomolecular Pathology and Intensive Care, University of Pisa, Pisa, Italy; ^3^ Department of General and Transplant Surgery, La Sapienza University of Rome, Rome, Italy; ^4^ University of Pisa Medical School Hospital, Pisa, Italy; ^5^ Department of Pathology, University of Pisa Medical School Hospital, Pisa, Italy; ^6^ Intensive Care Unit, University of Pisa Medical School Hospital, Pisa, Italy

**Keywords:** liver transplantation, carbon footprint, greenhouse gas emissions, CO2, pollution, global warming, energy consumption

## Abstract

**Background and aims:**

There is growing interest in the environmental impact of surgical procedures, yet more information is needed specifically regarding liver transplantation. This study aims to quantify the total greenhouse gas emissions, or carbon footprint, associated with adult whole-size liver transplantation from donors after brain death, including the relevant back-table graft preparation.

**Methods:**

The carbon footprint was calculated retrospectively using a bottom-up approach. This approach sums the volumes of energy consumption (kWh), volatile anesthetics (ml), solid waste (kg), and units of blood products transfused for each transplant. These consumption values were converted using validated conversion factors to the equivalent mass of carbon dioxide released into the environment (kg CO2e).

**Results:**

A total of 147 patients with a mean age of 55 years (male, 78.9%) who underwent liver transplants between 2021 and 2022 were analyzed, resulting in 45.5 tons CO2e. The mean (SD) carbon footprint for each procedure was 309.8 (33.2) kg CO2e [95% CI: 304.4; 315.3]. Total energy power consumption was 96.5 MW, contributing 65.4% of greenhouse emissions (29.8 tons CO2e), while volatile anesthetics, solid waste, and blood product transfusions contributed 8.0% (3.64 tons CO2e), 5.9% (2.7 tons CO2e), and 20.6% (9.4 tons CO2e), respectively. The duration of surgery (*t* = 29.0; *p* < 0.001), transfused red blood cells (*t* = 13.1; *p* < 0.001), fresh frozen plasma (*t* = 11.1; *p* < 0.001), platelets (*t* = 8.9; *p* < 0.001), and the use of an extracorporeal pump machine (*t* = 3.6; *p* < 0.001) had the greatest effects on greenhouse gas emissions.

**Conclusions:**

Liver transplantation requires significant energy and is associated with considerable greenhouse gas emissions, particularly during longer procedures. Transplant clinicians, hospital administrators, policymakers, and patients should be aware of the environmental impact of liver transplantation and collaborate to adopt sustainable energy practices.

## Introduction

Climate change is one of the biggest health threats of the 21st century (1), and all levels of society are encouraged to implement strategies to reduce greenhouse gas (GHG) emissions and protect humanity from rising temperatures (2). The health sector, a major service industry, has a significant carbon footprint (CF), with surgical, obstetric, and anesthesia care being major contributors (3, 4). Energy consumption associated with hospital activities significantly contributes to environmental pollution and emissions, including 12% acid rain, 10% GHG, and 10% air pollution (5). Operating rooms (OR) are three to six times more energy-intensive per square foot due to volatile anesthetics (VA) and their stringent heating, ventilation, and air conditioning (HVAC) requirements, lighting, patient monitoring equipment, and long hours of use (4, 6). Additionally, ORs contribute 20%–30% of hospital waste production, accounting for 70% of the 6,600 tons of waste produced by US hospitals daily, and packaging materials alone account for up to 40% of regulated medical waste from ORs (6). The global need to scale up care to meet patients' demands could further accelerate climate change if adaptation and mitigation measures are not implemented (3). In this regard, the evaluation of medical and surgical activities should incorporate environmental parameters in addition to the current quality assessment standards (3–6).

CF measures the total GHG emissions, both direct and indirect, that can be attributed to a process, product, institution, or industry (7). CF results in a quantifiable output expressed as the equivalent mass (in kilograms, kg) of CO2 released into the environment, known as CO2 equivalents (CO2e) (7). CF assessment methodologies are widely used in various industries, such as transportation, construction, manufacturing, and technology. However, their usage in the healthcare industry still requires improvement (8). There are three different methodologies for measuring the CF, but the simplest approach is a bottom-up analysis (8, 9). This is used for single standard procedures and evaluates the CF at one or a few locations, extrapolating the CF by multiplying it by the total number of procedures performed (8, 9). A broader way to examine the CF is through life-cycle analysis (LCA) (10). To achieve this, the movement of goods and services from various sectors of the economy into healthcare is carefully tracked. A monetary value is assigned to these flows; these are connected to the accounts of GHG emissions in each sector, and the carbon emissions from each of these inputs are finally attributed to the healthcare sector (8–10). A further approach is the so-called process-based LCA (8–10). This analysis defines the system boundaries from the production and transportation of medical supplies, transportation of patients and staff, energy usage in medical facilities, and waste produced by medical facilities (9). It covers the entire product or activity life cycle, from manufacturing to use and disposal (10). Its level of granular assessment makes it the most comprehensive approach for analyzing the environmental impact of a system (10).

Despite the increasing interest in the CF of medical and surgical activities (11–15), information on liver transplantation (LT) is still scanty. LT is a resource-intensive healthcare procedure that requires multiple teams, expensive equipment, sterilization processes, advanced surgical technologies, life support systems, and organ transportation (16). These activities consume significant energy and resources while producing a large amount of waste. Although the climate impact of complex surgical procedures like LT is generally accepted as necessary for treating patients with organ failure and ensuring quality care, it has been limitedly quantified or analyzed critically (9). This lack of evaluation is due to the complexity of LT procedures, which involve donor organ procurement, back table preparation, and implantation surgery. Additionally, there is limited awareness and information about the climate impact of surgical procedures despite surgeons being willing to implement changes to reduce energy consumption and CO2 production (17).

This study aims to quantify LT's CF using a bottom-up approach. This involves calculating the CO2e of energy consumed, VA, waste produced, and blood products transfused during the surgical procedure in an NHS hospital setting. The secondary aims are to raise transplant physicians' and surgeons' awareness of LT's climate impact and contribute to implementing actionable plans to reduce GHG emissions.

## Materials and methods

### Study design

This was a retrospective, single-center study at an Italian National Health System (NHS)-based liver transplant center.

### Study aims

The study's primary aim was to calculate the CF associated with LT and the preparation of back-table grafts. Its secondary aim was to develop actionable objectives for reducing CO2 emissions.

### Procedure boundaries

The activities included in the current study are transplantation surgery and back-table graft preparation. The reasons are: (a) donor surgery often occurs in different hospitals; (b) organs are transferred from procurement to transplant hospitals; (c) pre- and post-transplant patient care is extremely varied and can take place at the transplant hospital or with referring hepatologists.

### Patient population

To analyze the procedures initiated and completed in our ORs, this study required that participants be adults (18 years or older) receiving a full-size primary liver graft from a brain-dead donor between 2021 and 2022 and who underwent fast-track extubation after surgery. We excluded: (1) patients confined to the hospital, as their preoperative procedures are initiated in the intensive care unit (ICU); (2) intraoperative deaths; (3) recipients of split liver grafts since splitting is usually done *in situ* at the time of graft procurement; and (4) those transplanted from donors after cardiocirculatory death (DCD), as part of the graft exploration and preparation occurs in the hospitals where the donor is procured. Finally, we excluded procedures performed simultaneously with other transplants (e.g., kidney, pancreas) or non-transplant surgeries to isolate the OR power consumption associated only with liver transplantation (LT).

### Data source

For the current study, we used the administrative data from our institution's electronic OR database to register all surgical procedures. Due to the administrative nature of the data used in the current analysis, the study was exempt from approval by the local ethics committee per regional and national regulations.

### Anesthesia and surgical technique

The anesthesia technique has been described elsewhere (18): induction with intravenous (i.v.) fentanyl 0.2 mg, sodium thiopental, and cisatracurium, and maintenance with sevoflurane in a 50% air/oxygen low-flow respiratory mixture, remifentanil (0.2–0.3 μgkg^−1^min^−1^) and cisatracurium (3 μg kg^−1^min^−1^). Hemodynamic monitoring included invasive systemic arterial pressure and a pulmonary artery catheter (CCO/SVO2 Thermodilution Catheter, Edwards Life Sciences LLC, Irvine, CA, USA). A ROTEM device was always used to monitor intraoperative fibrinolysis [Werfen Instrumentation Laboratory SpA, Milan (I)]. An extracorporeal veno-venous bypass between the portal and inferior vena cava and the superior vena cava was used at the discretion of the surgical team. According to the surgeon's discretion, the biliary anastomosis was end-to-end choledoco-choledocostomy or bilio-enteric. A T-tube was used selectively based on liver graft quality and surgical anatomy. Ex-situ, ex-vivo machine perfusion (MP) was used selectively according to the donor's and recipient's clinical characteristics and the anticipated duration of cold ischemia time. During transplant surgery, the back-table graft preparation took place in a separate OR, which remained operational throughout the transplant procedure.

### Measure outcomes

**Figure 1:**
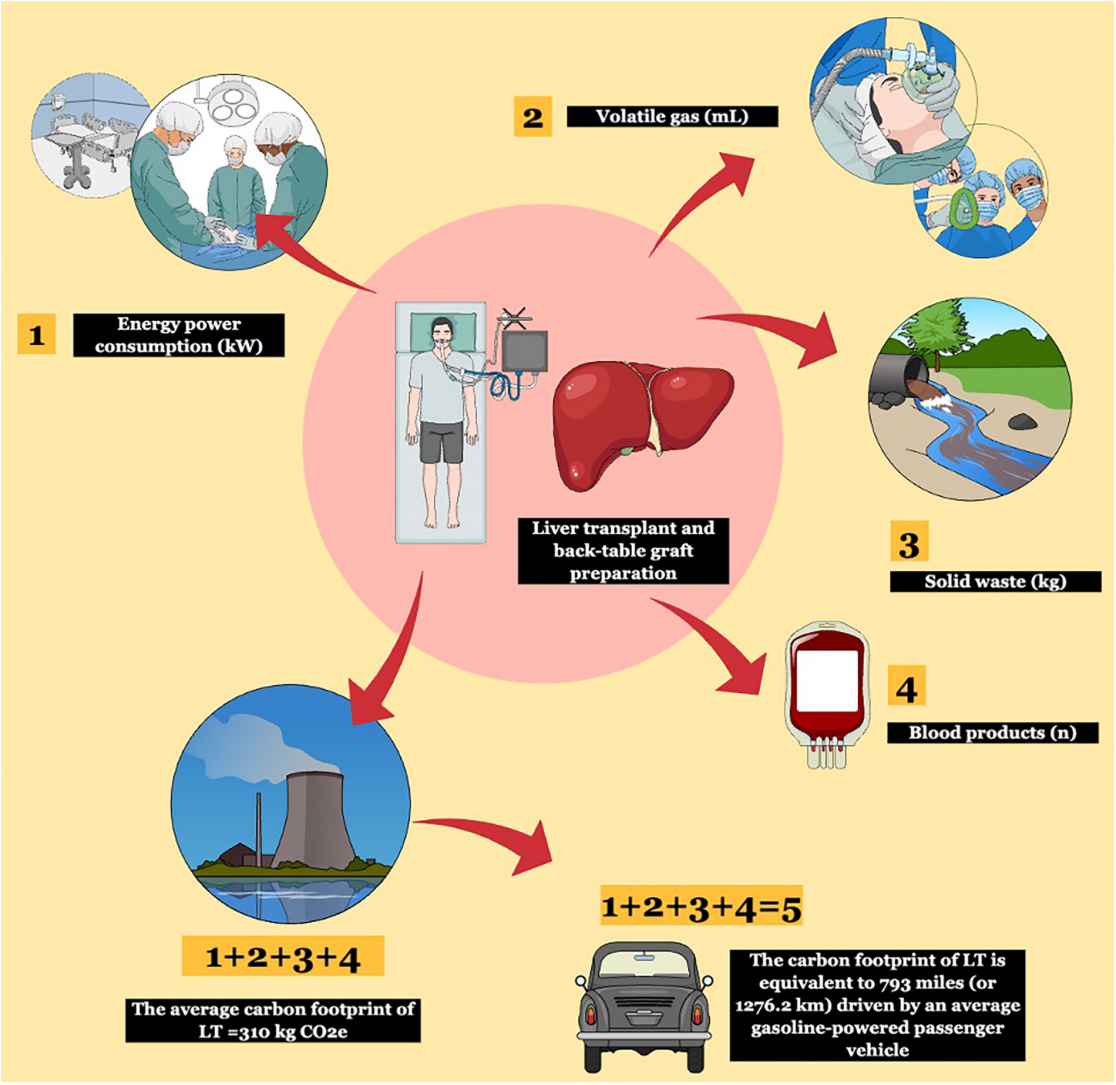
The bottom-up methodology used to calculate the carbon footprint (CF) of liver transplantation (LT). Four components were used for the bottom-up approach: energy power consumption, volatile gas, solid waste, and blood product transfusions. These were converted into kg CO2e according to validated conversion factors and the total CF was obtained. Using international calculators, greenhouse gas (GHG) emissions were later compared to human activities.

Our primary outcome was the cumulative GHG emissions, or CF, associated with LT and back-table graft preparation. This was calculated according to the bottom-up approach described elsewhere (8, 9) as the sum of (1) energy consumption, (2) VA (i.e., sevoflurane), (3) solid waste produced, and (4) units of blood products transfused for each surgical procedure of LT and associated back-table graft preparation (Figure 1).

Our analysis's standards, definitions, and assessment methodology comply with the British Standards Institute Publicly Available Specification 2050 (BSI PAS 2050) (19) and the Greenhouse Gas Protocol published by the World Business Council for Sustainable Development and the World Resources Institute (20).

Table 1 illustrates the theoretical assumptions for calculating energy consumption and solid waste during surgical procedures. Namely, consumption values were categorized into environmental, equipment, and instrument (Table 1), while solid waste was divided into infection control (drapes, gowns, gloves), consumables, sterile wrap, and single-use devices (Table 1). The Supplementary Materials (word and excel files) outline the operational definitions, standards, methods, and metrics used to calculate the CF. The energy conversion factor was obtained from the Italian *Istituto Superiore per la Protezione e la Ricerca Ambientale* (ISPRA) (21). The VA consumption and CF calculations were based on Biro et al. (22) and Wyssusek et al. (23), while the CF for blood units was derived from Hibbs et al. (24). Once obtained, the LT's CF values were converted into daily human activities using the equivalency calculator from the US Environmental Protection Agency (EPA) (25).

**Table 1:** Energy consumption and solid waste measurements used in the current study.

Purpose	Description	Assumptions
Energy		
Environmental	Heating/Filtration/Air exchange Anesthesia console OR lighting OR area lighting Recovery room lighting Nurse coordinator office lighting Relaxation area lighting Transplant nurse coordinator office area lighting.	• Energy per unit time from first nurse entry to last nurse exit. • Power consumption is derived from manufacturer and applied continuously as indicated above. • Data are derived from the Hospital Facilities Management and Engineering Department.
Equipment	Computers LCD/LED monitors Syringe pumps Infusion pumps Blood heater(s) Fridges/freezers Bair huggers Sequential compression devices Extracorporeal pump Machine perfusion PC	• Power consumption is derived from manufacturers’ information. • Computers, monitors are considered in use from first nurse entry to last nurse exit. • Pumps, heaters, bair huggers and compression devices are in use from patient entry till patient exit from the OR • Blood heaters are in use during surgery. • Fridges/freezers are running continuously 24/7. We used a 24-hour usage period. • Extracorporeal pump use and duration is recorded in the scrub tech report. • Machine perfusion use and duration is recorded by the scrub tech.
Instrument	Disposable energy-based surgical instrument Reusable energy-based surgical instrument	• Power consumption is derived from manufacturers’ information. • Use is considered continuous throughout surgery.
Solid waste		
Infection control	Drapes Gowns Gloves	• Drapes and gowns are made of Pluritex^TM^ fabrics intended for multiple uses (up to 100). • Double-glove technique for all sterile personnel. • Scrub tech shift changes are recorded in the electronic OR database. • Number of surgeons, scrub techs and assistants are derived from OR records and reports. • Total weight of gloves waste derived by measured weight of gowns multiplied by sterile personnel.
Consumables	Blue pack items (i.e., basins, sponges, towels, suction tubing, etc…)	• Blue pack weight was different for transplant vs. back-table surgery but was constant for all procedures. • Post-usage weight is recorded in OR reports.
Sterile wrap	Disposable blue wrap to maintain instrument tray sterility.	• Blue wrap weight is derived from manufacturer and number of trays is determined based on scrub tech OR records. • Post-usage weight is recorded in OR reports.
Single-use device	Single-use devices (e.g., skin staplers, disposable energy-based surgical instruments, etc…)	• Single-use device weight is derived from manufacturer's information. • Number of used devices is derived from OR scrub tech reports.

OR, operating room.

#### Statistical analyses

All personal (age, sex) and sensitive data (indication to transplant, date of surgery) associated with the surgical procedures used for the current analysis were anonymized according to the European Union's General Data Protection Regulation (GDPR) 279/2016. Based on their frequency and distribution, values are reported as means, standard deviations, medians, interquartile ranges (IQR), and frequencies. Continuous variables were compared via Student's *t*-test, Kruskal–Wallis test, or ANOVA methodology where appropriate. Multiple comparisons were controlled for via Bonferroni's method. As appropriate, select data are presented as bar charts, scatter, raincloud, and normal probability plots with 95% confidence intervals (CI).

After obtaining the per-procedure and total cohort CF, we tested the correlation between GHG emissions and clinical indicators of liver graft quality and complexity of surgery available in the OR records: recipient's age, sex, and indication to transplant; model for end-stage liver disease (MELD) score; donor's age and sex; donor's cause of death; duration of transplant surgery; cold ischemia time (CIT); warm ischemia time (WIT); extracorporeal pump machine (EPM); machine perfusion (MP); T-tube, and transfused blood products. These independent variables were initially tested with a univariate approach using Pearson's, Spearman's, point-biserial, or ANOVA tests, as appropriate. They were further used in linear regression analysis, and corresponding standardized effect sizes are shown. Co-linear variables were identified by means of Pearson's partial correlation analysis. All tests were held at the level of 0.05.

All statistical analyses and plots were run using the SPSS statistical package version 27.0 (SPSS Inc., Chicago, IL, USA). Plots were created with the DATAtab web-based application [DATAtab Team (2024). DATAtab: Online Statistics Calculator. DATAtab e.U. Graz, Austria. https://datatab.net]. This study conforms to the ethical guidelines of the 1975 Declaration of Helsinki as reflected in *a priori* approval by the institution's human research committee and was conducted according to the Strengthening the Reporting of Observational Studies in Epidemiology (STROBE) guidelines.

## Results

### Clinical and surgical characteristics

In the study period, 147 procedures met the inclusion criteria. The clinical and surgical characteristics of the study sample are shown in Table 2. Mean (SD) age at transplant was 55.0 (7) years [95% CI: 53.9; 56.2]; patients were predominantly male (78.9%) and the leading indications to transplantation were hepatitis B virus (HBV) chronic infection ± delta (HDV) infection in 30.6% of patients, metabolic dysfunction-associated steatotic liver disease (MASLD) in 29.2% of cases, and alcohol-related liver disease (ALD) in 14.9%. Hepatocellular carcinoma was present in 43 (29.2%) of patients. The mean (SD) lab MELD score at transplant was 13.5 (4.5) [95% CI: 12.8;14.2]. Donors were predominantly male (64.6%) with a mean (SD) age of 67.1 (15.2) [95% CI: 64.2; 69.9]. The cause of death was mainly cerebrovascular accident (CVA) (78.9%). The mean duration of transplant surgery was 6.5 (1.3) hours [95% CI: 6.3; 6.7]. Mean (SD) cold ischemia (CIT) and warm ischemia time (WIT) were 8.9 (0.9) hours [95% CI: 8.8;9.1] and 90.8 (9.1) [89.3;92.3] min, respectively. Transplantation was done with cava replacement and extracorporeal veno-venous circulation in 51.0% of cases, and a T-tube was used in 65.9% of patients. Five grafts (3.4%) underwent dual hypothermic perfusion (D-HOPE) before transplantation. Per-patient blood requirements consisted of a mean (SD) of 3.7 (1.5) [95% CI: 3.4;3.9] red blood cell (RBC) units, 4.3 (1.1) [95% CI; 4.1; 4.5] fresh frozen plasma (FFP) units, and a mean (SD) of 0.5 (1.1) [95% CI: 0.3; 0.6] units of platelets (PLT).

**Table 2:** Clinical and surgical characteristics of the study sample.

Variable	Mean (SD) [95% CI] (%)
Age at transplant, years	55.0 (7) [53.9;56.2]
Sex (male)	116 (78.9)
Indication to transplant	
MASLD	43 (29.2)
HBV	24 (16.3)
ALD	22 (14.9)
HBV-HDV	21 (14.3)
HCV	17 (11.6)
Met-ALD	8 (5.4)
PBC	3 (2.0)
Other	9 (6.1)
HCC (n)	43 (29.2)
Lab MELD	13.5 (4.5) [12.8;14.2]
Donor age, years	67.1 (15.2) [64.2;69.9]
Donor sex (male)	95 (64.6)
Donor cause of death (CVA) (*n*)	116 (78.9)
Duration of surgery (h)	6.5 (1.3) [6.3;6.7]
CIT (h)	8.9 (0.9) [8.8;9.1]
WIT (min)	90.8 (9.1) [89.3;92.3]
Cava-cava replacement (*n*)	75 (51.0)
T-tube (*n*)	97 (65.9)
RBC (*n*)	3.7 (1.5) [3.4;3.9]
FFP (*n*)	4.3 (1.1) [4.1;4.5]
PLT (*n*)	0.5 (1.1) [0.3;0.6]
T-tube (*n*)	97 (65.9)
MP^a^, *n*	5 (3.4)

ALD, alcohol-related liver disease; CIT, cold ischemia time; CVA, cardiovascular accident; FFP, fresh frozen plasma; HBV, hepatitis B virus; HCC, hepatocellular carcinoma; HCV, hepatitis C virus; HDV, hepatitis delta virus; MASLD, metabolic-dysfunction steatotic liver disease; Met-ALD, metabolic and alcohol-related liver disease; PBC, primary biliary cholangitis; PLT, platelet; RBC, red blood cell; WIT, warm ischemia time.

^a^ MP was dual-hypothermic in all cases.

### Consumption values

Table 3 displays the energy consumption values (in kW), the amount of VA used (in ml), the solid waste produced (in kg), and the number of blood product units transfused per procedure and for the entire cohort. Supplementary Table 1 illustrates the corresponding component values.

**Table 3:** Per-procedure mean energy consumption (kW), volatile anesthetics used (ml), solid waste produced (kg), and blood products units and corresponding total values for the current study sample.

Variable	Mean (SD) [95% CI]	Total (#147 procedures)
Energy (kW)	656.2 (72.6) [644.3; 668.0]	96,459.7
VA (ml)	124.1 (25.9) [119.9; 128.4]	18,245.7
Solid waste (kg)	22.8 (2.1) [22.5; 23.2]	3,355.7
Blood products (unit)	8.4 (2.5) [8.0; 8.8]	1,240

SD, standard deviation; VA, volatile anesthetic.

The mean (SD) power consumption per procedure was 656.2 (72.6) [95% CI: 644.3; 668.0] kW, for a total of 96.4 MW in the entire cohort. The mean (SD) sevoflurane consumption was 124.1 (25.9) [95% CI: 119.9; 128.4] ml for a total of 18.2 L. The mean (SD) solid waste amount per procedure was 22.8 (2.1) [95% CI: 22.5; 23.2] kg, i.e., a total of 3.3 tons for the whole study sample. The mean (SD) number of blood product units transfused per procedure was 8.4 (2.5) [95% CI: 8.0; 8.8], i.e., corresponding to a total of 1,240 units (Tab. 3). Supplementary Figure 1 illustrates the violin plot for the blood products consumed in the procedures of the current series.

### CF

**Figure 2:**
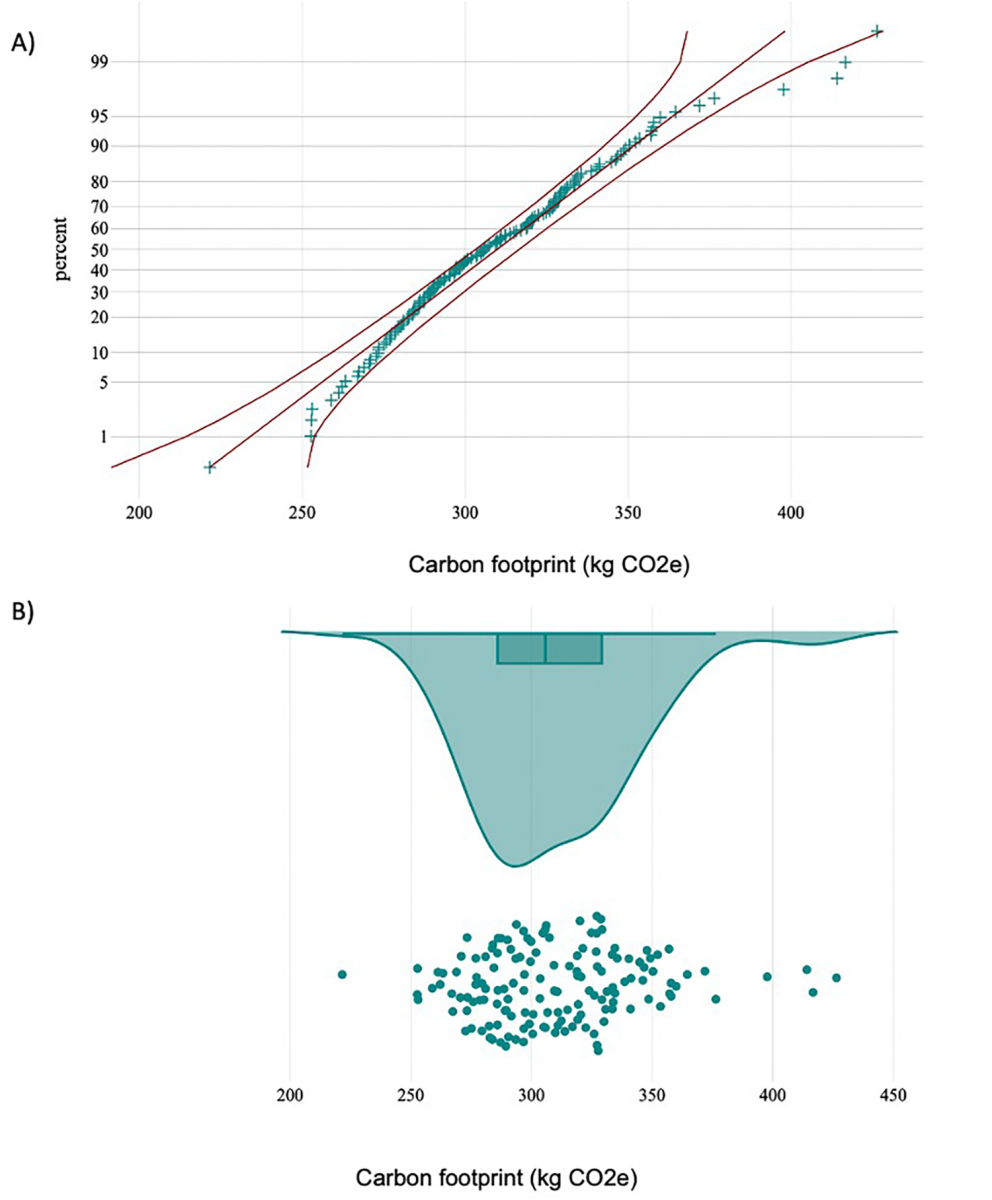
**(A)** normal probability plot with 95% CI of total greenhouse gas (GHG) emissions (kg CO2e) per liver transplant procedure. **(B)** Raincloud plot of total GHG emissions showing GHG per liver transplant procedure against the sample median and quartiles.

**Figure 3:**
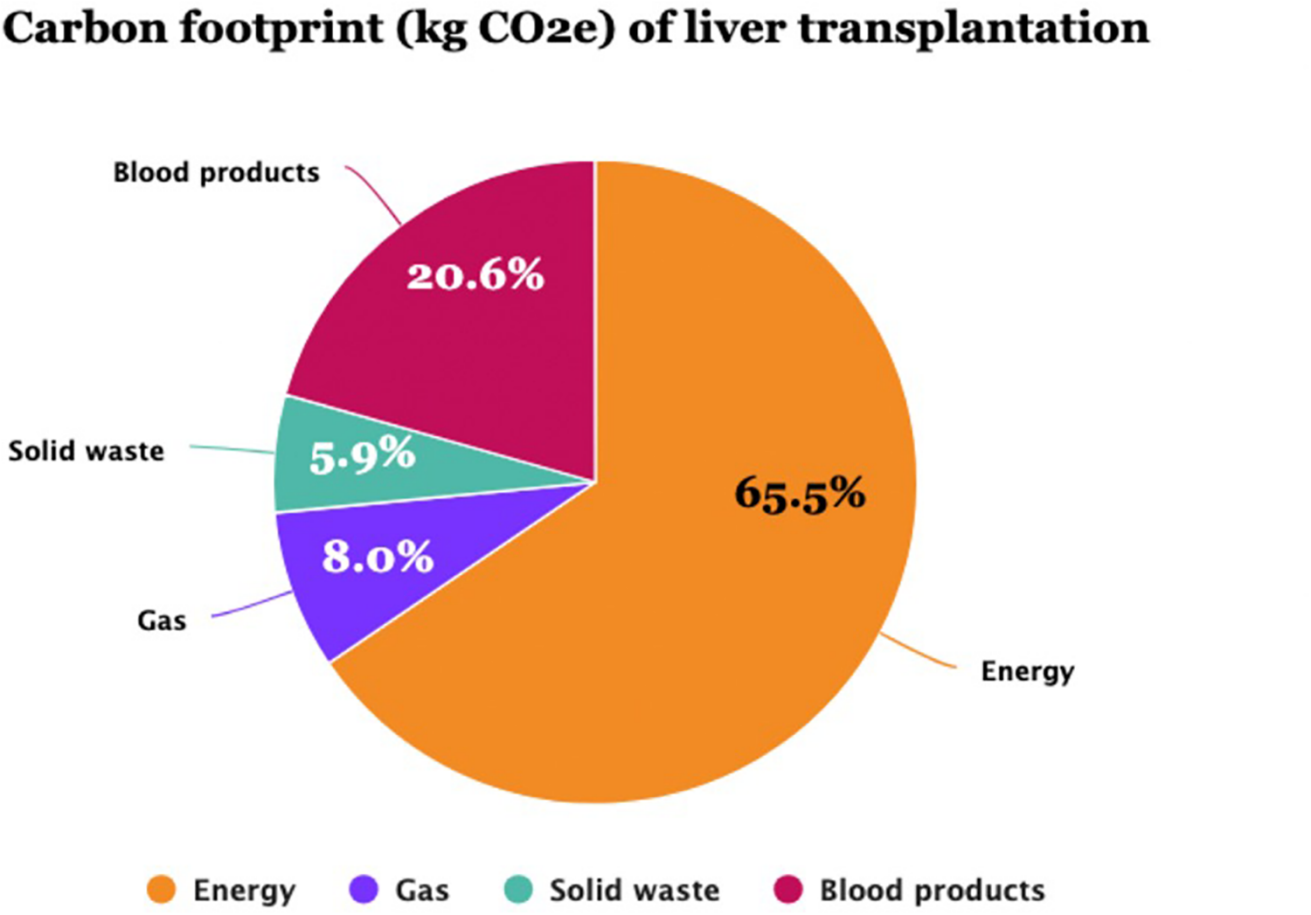
The carbon footprint (CF) components (kg CO2e) of liver transplantation in 147 procedures of the current study. Energy power accounted for the leading component of CF (65.5%), followed by blood product transfusions (20.6%).

**Figure 4:**
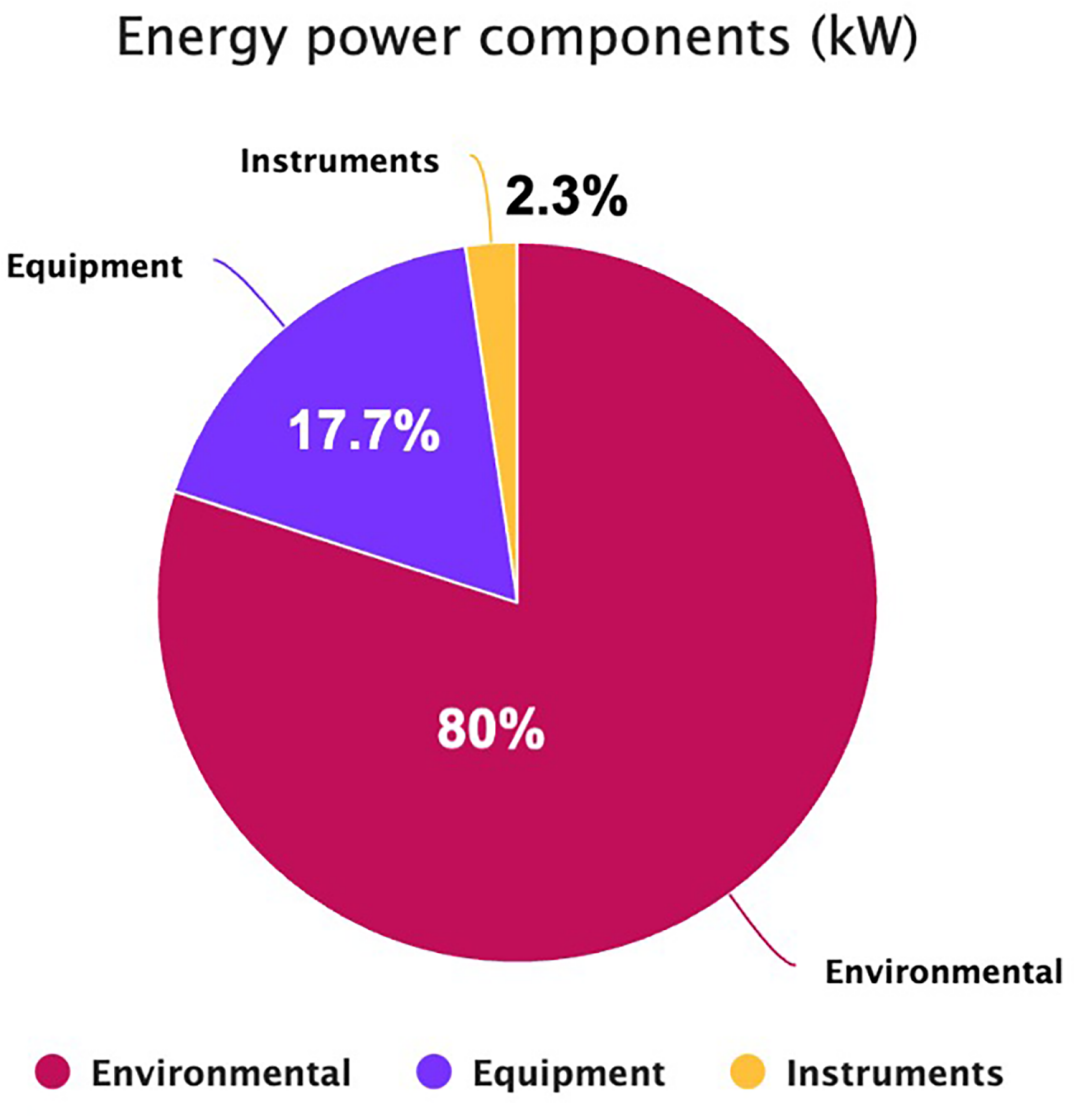
The energy power components (kW) of liver transplantation in 147 procedures of the current study. Environmental control devices accounted for the leading component of consumed electricity (80%), followed by equipment (17.7%) and surgical instruments (2.3%).

The per-procedure and overall CF values are illustrated in Table 4 and Figure 2. The mean (SD) CF of each LT procedure was 309.8 (33.2) [95% CI: 304.4; 315.3] kg CO2e. Energy consumption contributed 65.5% of total GHG emissions, with VA at 8.0%, solid waste at 5.9%, and blood products at 20.6% (Figure 3). Energy consumption was mainly due to environmental control devices, which accounted for 80%. Equipment and instruments contributed 17.7% and 2.3%, respectively (Supplementary Table 2; Figure 4).

**Table 4:** Per-procedure carbon footprint (kg CO2e) of energy consumed, volatile anesthetics, solid waste, and blood products transfusions and total values for the current study sample.

Variable	Mean (SD) [95% CI]	Total (#147 procedures) (%)
Per-procedure CF (kg CO2e)	309.8 (33.2) [304.4; 315.3]	45,537.7 (100)
Energy consumed (kg CO2e)	202.7 (22.4) [199.1; 206.4]	29,806.1 (65.5)
VA (kg CO2e)	24.8 (5.2) [23.9; 25.7]	3,649.1 (8.0)
Solid waste (kg CO2e)	18.4 (1.7) [18.1; 18.7]	2,708.1 (5.9)
Blood products (kg CO2e)	63.8 (18.7) [60.7; 66.8]	9,374.4 (20.6)

CF, carbon footprint; CO2e, carbon dioxide equivalent; SD, standard deviation; VA, volatile anesthetics.

Figure 2 illustrates the normal probability plot with a 95% CI (2A) for GHG emissions per procedure and the raincloud plot of each procedure's GHG emissions against the median and quartiles (2B). The entire cohort released 4.5 tons of CO2e.

The GHG emissions produced in an average procedure are equal to 793 miles (or 1,276.2 km) driven by an average gasoline-powered passenger vehicle and require 0.4 acres of US forest to be sequestered in one year (Figure 1).

### Correlation analysis

The results of the univariate correlation analysis are shown in Supplementary Tables 3 and 4. Duration of surgery (*p* < 0.001), RBC (*p* < 0.001), FFP (*p* < 0.001), and PLT (*p* = 0.006) were directly correlated with CF. Pearson's partial correlation analysis showed that the use of blood products lessened but did not eliminate the impact of the duration of surgery on CF (from r = 0.75 to r = 0.50).

The same independent variables were tested with linear regression analysis, as illustrated in Supplementary Table 4. Duration of surgery (*p* < 0.001), EPM (*p* < 0.001), RBC (*p* < 0.001), FFP (*p* < 0.001), and PLT (*p* < 0.001) were correlated with greater GHG emissions. The hierarchical order of the variables' effect sizes (ES) is illustrated in Supplementary Table 5. Duration of surgery had the greatest ES (*t* = 29.0) on CF, followed by RBC (*t* = 13.1), FFP (*t* = 11.2), PLT (*t* = 8.9), and EPM (*t* = 3.6).

## Discussion

### Study findings

In a selected sample of procedures and using a bottom-up approach, our study shows that LT is carbon-intensive, releasing a mean of 310 kg CO2e, and that energy consumption is the major contributor, accounting for 65.5% of GHG emissions, followed by blood products (20.6%) and anesthetic gases (8.0%) (Figure 3). Power and anesthetic gas consumption are due to LT being a lengthy procedure (mean 6.5 h) and requiring advanced technologies and equipment for environmental control because of the stringent requirements of ORs dedicated to transplant surgeries. Unlike other digestive surgeries, transfusion of blood products is common in LT due to portal hypertension-related pancytopenia and liver dysfunction-associated coagulation disorders (26).

While the comparability of our findings is currently impossible due to the lack of similar studies in the available literature, their transferability to clinical practice is hindered by some study limitations. First, our study was based on a retrospective review of surgical and administrative charts, which are not specifically designed to measure all the detailed components required for GHG emissions calculations. Appropriate methodologies should be implemented to define the process boundaries more accurately, to isolate the consumption values of procedures performed concomitantly with other surgeries (i.e., consumption shares), and to include regulated and contaminated waste while balancing the need for the safety of the study staff. Specific waste triage methodologies should be designed for the purpose of environmental studies, as they may be strictly dependent on the organization and structural requirements of hospital facilities. In our study, waste calculations were influenced by reusable gowns and drapes, which are not routine practices across different institutions.

Secondly, the calculated CF of LT was influenced by our organizational patterns, hospital architecture, and the type of electric energy sourcing. Our institution uses a second room for graft preparation due to OR capacity and to reduce CIT. Different models should be compared regarding their environmental efficiency, balancing the need to reduce prolonged CIT due to the expanding proportion of elderly donors (27). Furthermore, regional and national variations in the types and shares of energy sources (i.e., the percentage of solid fossil fuels, oil, natural gas, and renewable sources used for power production) deeply impact the final GHG emission values. These can greatly fluctuate across institutions, countries, and eras.

Finally, in the present series, we applied stringent selection criteria and included a limited number of adult cases to isolate procedures initiated and completed in our OR. Thus, we selected patients with less severe liver decompensation for whom we could not find any association between the clinical characteristics of donors and recipients and GHG emissions. However, to expand our knowledge on the environmental impact of LT, we must explore how GHG emissions fluctuate across the entire spectrum of transplant procedures, thus including more severe patients (i.e., acute liver failure cases), higher MELD scores, pediatric recipients, DCD grafts, living donor LT, and re-transplantations. The share of CO2 emissions related to donor organ procurement should also be incorporated using appropriate methodologies, considering the entire trajectory of donor and recipient surgery, including staff and graft transportation.

Modifying the clinical and organizational scenarios might shift the environmental impact of LT. Some factors, like those related to the clinical characteristics of the recipient population, can be modified to a limited extent, while others might yield greater change in GHG emissions. Reduction of the length of surgery is not always possible, especially in academic centers where the training of residents is an integral part of surgical procedures. On the contrary, surgical residents may be the preferred target of educational initiatives aiming at reducing GHG emissions from procedures. Optimization of blood transfusion might be pursued to reduce transfusion-associated morbidity rates, improve patient outcomes, and reduce the environmental impact of LT (26). Finally, while the contribution of solid waste was only 5.9% in our experience, it might increase considerably alongside power consumption for transplant procedures performed using a laparoscopic or robot-assisted approach (28, 29).

### The environmental impact of surgical procedures

Interpreting our findings within the context of available data is challenging because transplant surgeries are among the least studied procedures in environmental literature and complex digestive surgeries have been explored only to a limited extent (13, 14, 30–38). Our study confirms previous non-transplant reports highlighting that surgical operations are the most resource-intensive function in hospitals (4, 30–38). However, carbon emissions can vary widely based on the operation type, the level and source of electricity used, the types of anesthetic gases, the number of consumables involved, and the adopted calculation methodology (13, 14, 30–38). In previous reviews, the CFs of non-transplant procedures varied widely, ranging from 6 kg CO2e for cataract surgery to 814 kg CO2e for robotic endometrial staging and hysterectomy (13). Similarly, the CF was reported to be lower for tonsillectomy (7.5 kg CO2e) (33), skin cancer excision (28.5 kg CO2e) (14), and knee arthroplasty (85.5 kg CO2e) (33). In comparison, it was higher for meso-rectal excision (408.6 Kg CO2e) and cardiac surgeries (505.1 kg CO2e) (13). Depending on the procedure type, the environmental impact of its components varies significantly. Still, medical devices and consumables have been identified as the largest contributors to CF of minor surgeries, out-of-hospital procedures, and laparoscopic/robotic interventions, accounting for 73.3%–86.8% in recent studies (30–38). On the contrary, energy power was the greatest contributor to interventional radiology procedures (53.8%), while patient and staff transport was the largest contributor to skin cancer excisions (44.9%) (14, 30–38). Future studies on more intensive surgical procedures are needed to allow for comparisons with transplant surgery and streamline implementing environmentally friendly practices.

Recent reports have addressed kidney transplant care (39, 40), but information on LT is limited (41). A recent paper by Wall AJ et al. explored the CF of liver graft acquisition at one institution in the USA, showing that flight transportation of grafts and surgical teams resulted in 40.75 mT CO2e per month (41). The complexity of LT involves several phases - organ procurement, back-table graft exploration, and liver implantation - making it very challenging to calculate CO2e emissions. Moreover, organ procurement and transplant procedures often occur in different hospitals, leading to variations in energy-sourcing policies and sustainability practices. All these reasons may explain the delayed introduction of CF studies in the LT field.

### The proposal agenda

A wide range of green interventions have been advocated to reduce the CF of human activities (42) and the non-transplant surgical sector (43, 44), making a significant impact when implemented systematically (13, 44). The initiatives discussed in the literature cover all phases of surgical care, from patient referral to post-operative discharge and follow-up (44). They extend from reassessing the necessity of surgical care in daily clinical practice; redesigning preoperative care and surgical pathways in *ad-hoc* facilities with an integrated approach among surgeons, intensivists, and physicians, optimizing energy use in ORs; minimizing the use of harmful anesthetic gases; optimizing the utilization of medical and surgical products in ORs by applying circular economy principles and rationing, favoring reusable instruments over single-use ones, and extending their lifespan through repair, to include waste reduction, triaging, and recycling (44).

Since the secondary aim of this study was to establish actionable objectives that enhance our understanding of LT's CF and promote the sustainability of associated procedures, we propose a set of initiatives to implement environmentally friendly practices (Table 5). This proposal integrates international, national, and local strategies developed in non-transplant care by utilizing the holistic approach outlined by the UK Center for Sustainable Healthcare (44). It focuses on five areas aimed at raising awareness within the scientific community (16), aligning care with sustainable practices (12, 17, 26, 42, 45–52), supporting “green” initiatives (53, 54), and promoting a “green” transition among healthcare professionals, policymakers, administrators, patients, and stakeholders (55, 56) (Table 5).

**Table 5:** Proposed initiatives to improve the ecological sustainability of (liver) transplant care practices.

Area	Subarea	Actions	Challenges
Awareness		- Promote research, communication and scientific exchanges. - Standardize research methodologies with respect to the peculiarities of procurement and transplant surgeries.	- The most appropriate methodology is difficult to define due to the complexity of LT phases which incorporate procedures performed by multiple teams at different locations (16).
Care adjustment	Energy	- Promote eco-friendly practices and remove barriers to care changes (i.e., career incentives). - Hospitals should promote use of and investments in renewable energy sources. - Avoid energy power waste by behavioral changes, shutdown checklists and remote control.	- Carbon offset policies are necessary to achieve these goals (12). - Transition to renewable forms of energy supplies is to be supported at a national and international levels (45). - National governments are urged to subsidize carbon offset programs in public and private healthcare sectors (46).
	Waste	- Implement waste reduction initiatives. - Single-use instruments should be balanced against the ecologic impact of sterilization procedures. - Favor the acquisition and use of novel eco-friendly materials (i.e., biodegradable plastics) for packaging purposes. - Customize surgical packs to actual needs. - Enforce waste triage procedures. - Prolong the life cycle of reusable instruments.	- Local, regional and national authorities are urged to invest in and enforce waste recycling policies (circular economy) by way of detaxation and incentives (47). - Circular economy practices should be implemented in hospitals (circular healthcare) (e.g., repurposing of single-use surgical instruments and tools before their expiration; expand the life of surgical instruments by repair) but require interdisciplinary cooperation plans (48). - Resilience is required from healthcare professionals to implement circular economy practices (17).
	Anesthetic gases (49)	- Eliminate desflurane and favor sevoflurane. - Decommission central nitrous oxide piping. - Avoid use of nitrous oxide. - Minimize use of fresh gas flows.	- Continued education and training plans for OR and ICU staff is necessary (49).
	Blood product management (26)	- Address transfusion requirements right from the preoperative period. - Minimize iatrogenic blood losses during the entire transplant journey. - Correction of coagulopathy. - Improvement of anemia tolerance.	- ICU staff should be integrated in the preoperative patient selection/evaluation workup right from the start - Close patient monitoring is crucial (26).
	Organization	- Revise the organizational architecture by limiting the use of unnecessary rooms/spaces and service areas balancing efficacy, efficiency and safety (50). - Favor the use of green-energy vehicles for staff, patient and supply transportation (51, 52).	- Hospitals should invest in transplant coordinating teams with commitment to supervising the organization and workflow of procurement and transplant activities (52). - Transition to green mobility requires investments from hospitals, regional/national authorities and individuals (53).
Support/Leadership		- Implement and support “green” strategies in surgical teams and hospitals (54).	- Great variability exists across teams, hospitals, and authorities in their willingness to accept eco-friendly policies (55). - Stigma to practice changes is often hard to remove (55, 56).
Advocacy		- The transplant community should advocate a transfer to “green” strategies within institutions, authorities and scientific societies. - Scholars should foster interest in environmental research (56).	- The scientific relevance of environmental research has to be promoted (56).

At a regional or national level, the most effective initiatives consist of carbon-reducing interventions shifting energy shares from solid fossil to renewable sources, including solar and wind energy technologies. However, this share can be used in healthcare facilities only when the energy source is available in satisfactory quantities located near to them (57). To be more effective, energy sourcing policies should be combined with interventions specific to architecture such as thermal insulation for buildings, the adoption of carbon-efficient heating and cooling systems, the utilization of energy-efficient vehicles and devices, and strategies for conserving energy related to lighting and managing energy-intensive appliances (57).

Within hospitals, transplant surgery-specific interventions pertain to behaviors and practices in ORs alongside what has been reported for non-transplant procedures. Waste disposal in ORs is one such area, and existing studies have already shown that substantial savings are achievable with proper segregation and recycling practices (58). These initiatives include increasing the number of bins, placing identification labels above them, and providing education for staff along with clear hospital guidelines (59). Another strategy is to reduce the percentage of contaminated waste, as approximately 66% of OR waste is inappropriately contaminated during surgery (59). A recent report from Italy reveals that 57% of waste is disposed of improperly and 71% could have been recycled (60). Additionally, the preoperative phase generated the largest amount of waste (48%) and had the highest percentage of incorrect differentiation (72%) (60).

Although there is no specific study available in the transplant setting, recycling waste, surgical packaging, and instruments is another strategy to reduce GHG emissions intensity. Surgical instrument wraps can be diverted from general waste to recycling streams with appropriate initiatives at local ORs, reducing CO2e emissions and cost savings (61). Due to the blood transfusion requirements of LT surgery, it might be interesting to note that a recent German study has demonstrated the feasibility of recycling complex and contaminated disposable surgical instruments, resulting in 239 kg of material being recycled over six months and a reduction of 545 kg CO_2_e (62). Recycling involved a minimal additional workload of less than five minutes but required coordination with government authorities and was approximately 3.9 times more expensive than incineration due to the higher recycling costs and the need for in-house decontamination (62). Reusing and repairing surgical instruments rather than replacing them can reduce environmental and financial costs (63, 64). However, the extent to which repair may play a role in mitigating the environmental impact of other surgical instruments has not yet been elucidated (64). Energy efficiency in the surgical environment can be enhanced by optimizing surgical trays, decreasing their preparation, decontamination, and processing times (64); repurposing underused instruments for various specialties, institutions, or countries (64), and investing in innovative technologies that utilize energy-efficient materials (65). While reusable instruments provide notable benefits from both medical and economic viewpoints, there is limited data regarding their ecological impact; however, the existing information clearly supports the use of reusable instruments (66). In the only retrospective comparative study available, utilization of reusable surgical instruments (scissors, trocars, and staplers) was associated with a 75% reduction in CF (67). This information can be incorporated to develop environmentally friendly initiatives in LT, expanding to technologies and instruments specific to the transplant setting.

Transforming hospital organizations and behaviors poses significant challenges (68). Various barriers impede the evolution of care relating to individuals (e.g., knowledge, skills, and attitudes), institutions (e.g., budgets, strategies, and readiness), geography/infrastructure (e.g., infrastructure and public awareness), politics (e.g., regulations and incentives), and stakeholders (e.g., patient awareness and knowledge) (68). However, transformational leadership, characterized by a clear vision and a collaborative approach, has been identified as a key factor for success (68).

Finally, both national and international strategies have been proposed to support the green transition in hospitals through carbon offset policies and incentives (57, 69). Some authors have suggested implementing nature-based solutions, such as forest conservation, to combat climate change and encourage healthcare organizations to participate in either large compliance or small voluntary markets (69). Various incentives and schemes for reducing GHG emissions, including carbon taxes, carbon trading, and carbon offsets, have been suggested (69, 70). In the healthcare sector, however, the most suitable strategy depends heavily on local and national regulations, as well as the hospital's financial structure. We advocate for smaller voluntary markets, where hospitals and healthcare professionals are encouraged to reduce their GHG emissions from transportation, electricity use, and other sources. The resulting clean energy investment plans may be financed through internal, external, or mixed resources, depending on the specific project's scale and nature. The role of central authorities is crucial in facilitating these schemes, including extending payback periods (57).

### Conclusive remarks

This first attempt to measure the carbon footprint of LT used a bottom-up methodology. We found that energy production is the major contributor to CO2 emissions, followed by blood product transfusions. These results arise from the surgery duration and the blood transfusion needs of LT recipients and are influenced by the energy sources mix used in our country. Additionally, broader initiatives are needed at both national and international levels to benchmark the carbon footprint of organ procurement and transplantation procedures. Transplant physicians, surgeons, administrators, policymakers, and patients must recognize the environmental impact of transplant activities and collaborate to implement more sustainable transplant care models.

## Data Availability

The raw data supporting the conclusions of this article will be made available by the authors, without undue reservation.
