# Supplementary material for: The carbon footprint and energy consumption of liver transplantation
Source: Front Transplant. 2025 Jan 6;3:1441928. doi: 10.3389/frtra.2024.1441928 (PMC11743657; doi:10.3389/frtra.2024.1441928)

THE CARBON FOOTPRINT AND ENERGY CONSUMPTION OF LIVER TRANSPLANTATION

SUPPLEMENTARY MATERIALS

Materials and Methods

A) ENERGY CONSUMPTION

Energy consumption was calculated using the Italian electric network's energy mix and a location-based approach. The Italian national electricity network utilizes gas, coal, and photovoltaic (renewable) energy. It is, therefore, impossible to determine the exact source of the electric energy used in the OR. For the current study, we have used data from the national authority ISPRA (*Istituto Superiore per la Protezione e la Ricerca Ambientale*) (1). Based on their report, the national energy mix consists of solid fossil fuels for about 6%; oil and petroleum products for about 30%; natural gas for about 40%; renewables and biofuels for about 20%, and the remaining share (less than 4%) was composed of electricity and nonrenewable waste. The conversion factor provided by ISPRA for 2022 was 0.309 kg CO2e/kW (1).

The energy power characteristics of all the used appliances were derived from the manufacturers' specifications. Equipment, instruments, and appliances were categorized as illustrated in Table 1. Once obtained, energy consumption in kWh was converted to kg CO2e using the 2022 conversion factor provided.

References

1. Istituto Superiore per la Protezione e la Ricerca Ambientale (ISPRA) official website. (www.isprambiente.gov.it). Accessed on April 1st, 2024.

B) VOLATILE ANESTHETICS

Sevoflurane consumption was derived retrospectively from anesthesiologic records and calculated according to the formula suggested by Biro (1):

*Fluid VA = specific weight * Avogadro's gas constant * (273 + temperature)/molecular weight * 273*

Where the specific weight for sevoflurane is 1.53 g/mL, and Avogadro's gas constant states that at a standard atmospheric pressure of 760 mmHg (at sea level) and a temperature of 0°C = 273°K, one mole of any gas consists of 6.023 x 10^{23} molecules which in turn occupy a volume of 22'400 mL. The temperature of the vaporizer is close to room temperature. Due to the loss of energy during evaporation, there is a tendency to cool the vaporizer, which is counteracted by its inbuilt high-temperature conductibility. It is therefore suggested to subtract 2°C from the prevailing room temperature, and the result will be added to 273°K. The molecular weight of sevoflurane is 200 g/mol.

Once the sevoflurane consumption was obtained, it was converted into CO2e based on what Wyssusek et al. indicated and derived from the gas's global warming potential over 100 years: 250 mL of sevoflurane has a CO2e of 49Kg (i.e., 0.196 kg CO2e/mL) (2).

References

1. Biro P. Calculation of volatile anaesthetics consumption from agent concentration and fresh gas flow. Acta Anaesthesiol Scand 2014 Sep;58(8):968-72. doi: 10.1111/aas.12374.
2. Wyssusek K, Chan KL, Eames G, Whately Y. Greenhouse gas reduction in anaesthesia practice: a departmental environmental strategy. BMJ Open Qual 2022;11(3):e001867. doi: 10.1136/bmjoq-2022-001867.

C) SOLID WASTE

The amount of landfill solid waste produced was measured based on the official OR charts. Waste was categorized by production source and quantified as described in Table 1 into infection, sterile wraps, consumables, and single-use devices. Waste production was determined based on OR instrument data specific to each recipient and back-table surgery procedure. Due to variations in CO_2 emissions according to the type of solid waste, we used a conversion factor of 0.807 (kg CO_2e/kg, i.e. 1 kg of waste produces 0.807 (807g) of CO_2e), as indicated in previous reports from Italy (1). Due to the potential release of blood and pathogens when manipulated, regulated waste (such as liquid or semi-liquid blood, blood-soaked, pathological, microbiological waste, and contaminated sharps) was excluded from the bottom-up analysis (2). We used data on the CF of RBC (red blood cells) to compensate for this (3).

References

1. Istituto Superiore per la Protezione e la Ricerca Ambientale (ISPRA) official website. (www.isprambiente.gov.it). Accessed on April 1st, 2024.
2. Rodríguez-Jiménez L, Romero-Martín M, Spruell T, Steley Z, Gómez-Salgado J. The carbon footprint of healthcare settings: A systematic review. J Adv Nurs 2023;79:2830-2844. https://doi.org/10.1111/jan.15671
3. Hibbs SP, Thomas S, Agarwal N, Andrews C, Eskander S, Abdalla AS, Staves J, Eckelman MJ, Murphy MF. What is the environmental impact of a blood transfusion? A life cycle assessment of transfusion services across England. Transfusion 2024 Apr;64(4):638-645. doi: 10.1111/trf.17786.

BLOOD PRODUCTS

The CF of transfused blood products was calculated based on a recent publication by Hibbs et al. (1). Using an LCA approach, the authors demonstrated that each RBC unit corresponded to the release of 7.56 kg CO2e. We applied this value to all blood products since no reference exists for FFP and PLT transfusions.

References

1. Hibbs SP, Thomas S, Agarwal N, Andrews C, Eskander S, Abdalla AS, Staves J, Eckelman MJ, Murphy MF. What is the environmental impact of a blood transfusion? A life cycle assessment of transfusion services across England. Transfusion 2024 Apr;64(4):638-645. doi: 10.1111/trf.17786.

The CF of each procedure was calculated using 49 items (Supplementary excel file). The cumulative CF of LT (transplant + back-table surgery) was finally calculated as follows:

*CF (kg CO2e) = power consumption (kWh) * 0.309 kg/kW + VA consumption (mL) * 0.196 kg/mL + solid waste (kg) * 0.807 kg/kg + units blood products * 7.56 (units/kg)*

To assess the co-linearity among independent variables (blood products and length of surgery), we used Pearson's partial correlation analysis.

This was as follows: x= CF; y=time; z=blood products

r_{xy} = 0.75

r_{xz}= -0.04

r_{yz} = 0.56

This analysis shows that the length of surgery has a far greater impact on CF (0.75) than the use of blood products (-0.04).

To exclude the impact of blood products on CF of surgical procedures, we used the partial correlation formula:

$$r_{xy,z} = \frac{r_{xy} - r_{xz} \cdot r_{yz}}{\sqrt{(1 - r_{xz}^2) \cdot (1 - r_{yz}^2)}}$$

The result is 0.505.

This means that removing the impact of "blood products" lessens, but does not eliminate, the correlation between the duration of surgery and CF.

<u>Supplementary Table 1</u>: Per-procedure mean energy consumption (kW), volatile anesthetics used (mL), and solid waste produced (kg) and total values for the current study sample.

**Variable**	**Mean (SD) [95% CI]**	**Total (#147 procedures)**
Energy (kW)	656.2 (72.6) [644.3; 668.0]	96459.7
Environmental	*524.5 (52.9) [515.8;533.1]*	*77100.6*
Equipment	*115.9 (12.4) [113.9;117.9]*	*17043.4*
Instrument	*15.0 (3.1) [14.5;15.5]*	*2208.5*
VA (mL)	124.1 (25,92) [119.9;128.4]	18245.7
Solid waste (kg)	22.8 (2.1) [22.5;23.2]	3355.7
Infection	*5.9 (0.66) [5.8;6.0]*	*875.1*
Sterile wrap	*1.86 (0.24) [1.8;1.9]*	*273.6*
Single-use device	*5.3 (0.6) [5.2;5.4]*	*778.4*
Consumables	*9.7 (1.79) [9.4;10.0]*	*1428.6*
Blood products (Units)	8.4 (2.5) [8.0;8.8]	1240

NOTE: SD, standard deviation; VA, volatile anesthetic.

<u>Supplementary Table 2</u>: Per-procedure carbon footprint (kg CO2e) of energy consumed, volatile anesthetics, solid waste, and blood product transfusions and total values for the current study sample.

Variable	Mean (SD) [95% CI]	Total (#147 procedures)
Per-procedure CF (kg CO2e)	309.8 (33.2) [304.4;315.3]	45537.7
Energy consumed (kg CO2e)	202.7 (22.4) [199.1;206.4]	29806.1
Environmental	*162.1 (16.4) [159.4;164.7]*	*23824.1*
Equipment	*35.8 (3.8) [35.2;36.4]*	*5266.4*
Instrument	*4.6 (0.9) [4.5;4.8]*	*682.5*
VA (kg CO2e)	24.8 (5.2) [23.9;25.7]	3649.1
Solid waste (kg CO2e)	18.4 (1.7) [18.1;18.7]	2708.1
Infection	*4.8 (0.5) [4.7;4.9]*	*706.2*
Sterile wrap	*1.5 (0.2) [1.4;1.5]*	*220.8*
Single-use device	*4.3 (0.5) [4.2; 4.3]*	*628.2*
Consumables	*7.8 (1.4) [7.6;8.0]*	*1152.9*
Blood products transfusions (kg CO2e)	63.8 (18.7) [60.7;66.8]	9374.4

NOTE: CF, carbon footprint; CO2e, carbon dioxide equivalent; SD, standard deviation; VA, volatile anesthetics.

<u>Supplementary Table 3:</u> Univariate correlation analysis between clinical and surgical independent variables and greenhouse gas (GHG) emissions. The variables that were correlated with GHG emissions were duration of surgery, red blood cells, fresh frozen plasma, and platelet transfusions.

Variable	r/F	p
Recipient's age at transplant (years)	0.08	0.327
Recipient's sex (male)*	0.02	0.817
Indication to transplant**	0.94	0.501
MELD	0.07	0.394
Donor age (years)	0.06	0.484
Donor sex (male)*	0.07	0.433
Donor CVA (yes/no)*	0.15	0.074
Duration of surgery (hrs)	0.75	<0.001
CIT (hrs)	0.01	0.927
WIT (min)	0.06	0.5
EPM (yes/no)	0.11	0.18
MP (yes/no)	0.06	0.438
T-tube (yes/no)	0.54	0.463
RBC (units transfused)	0.42	<0.001
FFP (units transfused)	0.44	<0.001
PLT (units transfused)	0.23	0.006

NOTE: CF, carbon footprint; CO2e, carbon dioxide equivalent; HVAC, heating, ventilation and air conditioning; RBC, red blood cells units transfused; SD, standard deviation; VA, volatile anesthetic. *Point-biserial correlation; **ANOVA.

<u>Supplementary Table 4:</u> **Multivariate regression analysis of clinical predictive variables of carbon footprint.** The clinical co-variates that showed a significant association with carbon footprint were duration of surgery, red blood cell transfusion, fresh frozen plasma, platelets transfusion, and extracorporeal pump machine.

	**Unstandardized Coefficients**	**Standardized Coefficients**		**95% confidence interval for B**	
Model	B	Beta	Standard error	lower bound	upper bound
(Constant)	126.7		14.3	98.3	155.0
Sex F	0.8	0.01	2.1	-3.4	5.1
Age at tx	-0.01	0	0.1	-0.3	0.2
Indication HCV	-0.9	-0.01	2.9	-6.6	4.7
Indication HCV-ALD	1.5	0.01	6.2	-10.7	13.7
Indication HBV	-0.4	0	2.6	-5.7	4.8
indication ALD	2.9	0.03	2.8	-2.5	8.4
Indication MET-ALD	0.6	0	4	-7.3	8.6
Indication HBV-HDV	0.3	0	2.8	-5.2	5.8
Indication PBC	-6.8	-0.03	5.3	-17.4	3.7
Indication HCV-HBV	0.2	0	7.2	-13.9	14.5
Indication OTHER	-4.7	-0.02	7.2	-18.9	9.5
Duration of surgery	18.8	0.8	0.6	17.5	20.1
CIT	-0.2	0	0.9	-2.1	1.7
WIT	-0.1	-0.04	0.1	-0.3	0.05
MP	4.5	0.02	4.8	-4.9	14.0
MELD	-0.01	0	0.2	-0.4	0.4
Donor age	0.02	0.01	0.05	-0.08	0.1
Donor sex F	2.6	0.04	1.9	-1.2	6.4
Donor death CVA 0	-0.2	0	2.2	-4.5	4.1
EPM	0.7	0.09	0.2	0.3	1.1
RBC	7.6	0,3	0.6	6.5	8.8

	Unstandardized Coefficients	Standardized Coefficients		95% confidence interval for B	
Model	B	Beta	Standard error	lower bound	upper bound
FFP	8.7	0.3	0.8	7.2	10.3
PLT	7.1	0.2	0,8	5.5	8.7
HCC	3.3	0.04	1.9	-0.55	7.1

NOTE: ALD, alcoholic liver disease; CVA, cerebrovascular accident; EPM, extracorporeal pump machine; FFP, fresh frozen plasma; HBV, hepatitis B virus; HCV, hepatitis C virus; HDV, hepatitis delta virus; MASLD, metabolic dysfunction-associated steatotic liver disease; MELD, model for end-stage liver disease; MET-ALD, metabolic dysfunction and alcohol-associated liver disease; MP, machine perfusion; PBC, primary biliary cholangitis; PLT, platelets; RBC, red blood cells.

<u>Supplementary Table 5:</u> hierarchical order of variables' effect sizes associated with carbon footprint of liver transplant procedures. Duration of surgery showed the greatest effect size (t=2.49), followed by red blood cell transfusions (t=13.3), fresh frozen plasma (t=11.2), platelets transfusions (t=8.7), extracorporeal pump machine (t=3.4), hepatocellular carcinoma (t=1.7) and donor female sex (t=1.35). However, these last two did not achieve statistical significance.

**Model**	**t**	**p**
Duration of surgery	29.5	<0.001
RBC	13.3	<0.001
FFP	11.2	<0.001
PLT	8.7	<0.001
EPM	3.4	0.001
HCC	1.7	0.09
Donor sex F	1.3	0.2
Indication ALD	1.1	0.3
MP	0.9	0.3
Donor age	0.4	0.7
Sex F	0.4	0.7
Indication HCV-ALD	0.2	0.8
Indication MET-ALD	0.2	0.9
Indication HBV-HDV	0.1	0.9
Indication HCV-HBV	0.04	0.9
Age at tx	-0.05	0.9
MELD	-0.07	0.9
Donor death CVA	-0.08	0.9
Indication HBV	-0.2	0.9
CIT	-0.2	0.8
Indication HCV	-0.3	0.7
indication OTHER	-0.7	0.5
Indication PBC	-1.3	0.2
WIT	-1.5	0.1

Supplementary Figures

<u>Supplementary Figure 1:</u> **Violin plot of per-procedure blood product consumption in the present series.** RBC, red blood cells; FFP, fresh frozen plasma; PLT, platelets.

Supplementary Figure 1

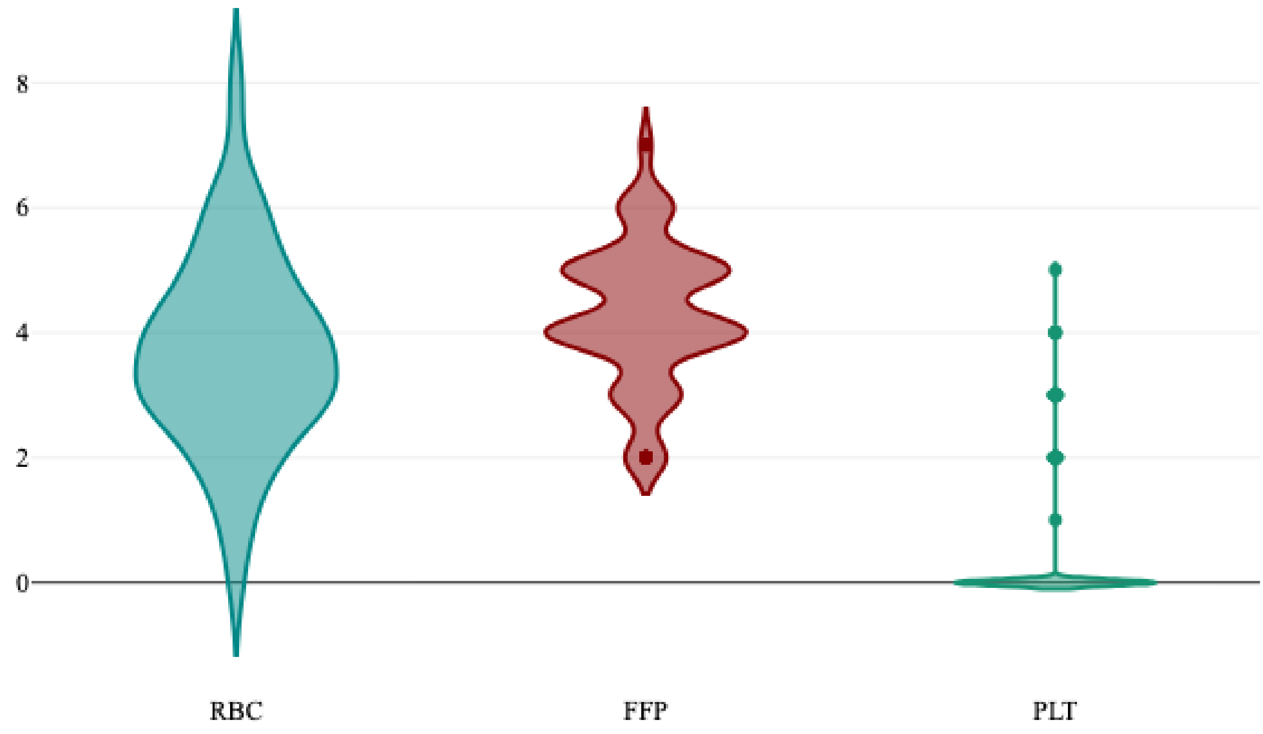

Supplement: Supplementary file 1 [file Datasheet1.pdf]
